# Presenteeism among healthcare professionals in Mongolia: A mixed-method multicenter study

**DOI:** 10.3934/publichealth.2026040

**Published:** 2026-07-13

**Authors:** Oyundari Batsaikhan, Odgerel Chimed-Ochir, Delgerjargal Dorjbal, Erdmaa Tserendondov, Otgontogoo Oidovsuren, Yumiya Yui, Ami Fukunaga, Yoshihisa Fujino, Bayasgalanmunkh Baatar, Tatsuhiko Kubo

**Affiliations:** 1 Department of Public Health and Health Policy, Hiroshima University, Hiroshima, Japan; 2 Department of Rehabilitation Medicine, Fujita Health University, Toyoake, Aichi, Japan; 3 Darkhan-Uul Medical School, Mongolian National University of Medical Sciences, Darkhan-Uul province, Mongolia; 4 Department of Environmental Epidemiology, University of Occupational and Environmental Health, Kitakyushu, Japan

**Keywords:** presenteeism, occupational health, healthcare personnel, Mongolia, pilot project

## Abstract

**Background:**

Presenteeism, defined as attending work while ill, is a growing concern among healthcare professionals due to its impact on well-being, work function, and the quality of care. Despite extensive global research, evidence from Mongolia remains limited, where workforce shortages and systemic constraints uniquely shape presenteeism. We examined factors associated with presenteeism among Mongolian healthcare professionals.

**Methods:**

This study used an explanatory sequential mixed-method design. The quantitative phase consisted of a cross-sectional online survey of physicians and nurses at three referral-level state hospitals, who were selected by convenience sampling. The qualitative phase consisted of semi-structured interviews with clinician managers, selected through purposeful sampling until thematic saturation was reached. Data were thematically analyzed, and the findings were integrated through triangulation.

**Results:**

A total of 465 survey respondents (physicians and nurses) participated in the quantitative phase, of which 64.5% reported presenteeism. The factors significantly associated with presenteeism were psycho-physical health complaints (AOR = 4.51, p < 0.001), coworkers' support (AOR = 2.38, p < 0.001), and workload (AOR = 2.08, p < 0.001). Qualitative analysis of 14 interviews with clinician managers identified five interrelated themes: inadequate occupational health policy, shortcomings of occupational infrastructure, staff shortages and heavy workloads, informal workplace practices and professional norms, and unaddressed health burdens. Integration of quantitative and qualitative findings indicates that these associations are shaped by underlying organizational and structural conditions. Qualitative data explain how heavy workload, staff shortages, and informal workplace norms compel healthcare professionals to work despite illness, while strained coworker relationships reflect peer pressure and team-based expectations. Taken together, similar to the existing global literature, the results reveal that structural and organizational challenges, rather than individual health-related behaviors, are the primary drivers of presenteeism in this context.

**Conclusions:**

The findings suggest that addressing presenteeism among healthcare professionals in Mongolia and similar low-resource settings requires coordinated national efforts targeting workplace conditions, organizational support, and occupational health policies to promote workforce well-being.

## 1. Introduction

Presenteeism, defined as working despite poor physical or mental health, has become a major concern in occupational and public health. Working while ill can impair employee well-being and work function, negatively affect job performance, and disrupt institutional functioning. Employees who work unwell are often less productive and more prone to errors, accidents, and near-miss events [1]. In healthcare settings, the consequences are particularly serious, as presenteeism affects not only healthcare professionals but also the quality and safety of patient care and overall institutional performance [2]. As awareness of its impact has grown, the conceptualization of presenteeism has evolved. Earlier studies primarily focused on sickness presenteeism, whereas more recent research highlights broader workplace determinants, including biological, ergonomic, and psychosocial hazards, as well as organizational culture and management practice [3]. Consequently, understanding the causes and addressing the consequences of presenteeism in healthcare settings has become increasingly important worldwide.

Despite extensive research in high-income countries, evidence from low-income regions remains limited, and the structural and cultural determinants are poorly understood [4]. These gaps are evident in Mongolia, where features of the health system may exacerbate presenteeism. The Mongolian health system, based on the Semashko model, is characterized by centralized hospital-based services and hierarchical management. These structural features, combined with vast geography, sparse population, and harsh climate, challenge workforce sustainability and equitable service delivery. Although universal healthcare coverage exists, shortages of healthcare professionals, limited rural resources, and excessive urban workloads persist [5]. Although medical personnel are the backbone of the system, insufficient attention is given to occupational health and protection.

Previous occupational health research in Mongolia primarily focused on physical and mental health [6]–[8] or broader issues such as working conditions and workforce distribution [9], but evidence on presenteeism remains extremely limited. This study was conceptually informed by the Job Demands–Resources Model (JD-R model), a widely used framework for understanding how workplace conditions influence employee well-being and performance. The model proposes that excessive job demands (e.g., heavy workloads and time pressure) combined with insufficient job resources (e.g., organizational support, staffing, and leadership) can lead to strain and health problems. Because presenteeism often occurs when employees continue working despite illness under demanding work conditions, the JD-R model provides a useful framework for examining its organizational determinants in healthcare settings. Accordingly, this study aimed to identify individual, organizational, and sociocultural factors associated with presenteeism to inform workforce management, occupational health policies, and strategies to enhance the well-being and resilience of healthcare professionals in Mongolia.

## 2. Materials and methods

### 2.1. Study design

An explanatory sequential mixed-method design was employed to examine the prevalence of presenteeism and its determinants among physicians and nurses in Mongolia (Supplementary Table S1). First, a pilot quantitative cross-sectional survey was conducted to explore the preliminary factors associated with presenteeism. This was followed by a quantitative phase with a subsequent qualitative phase to provide in-depth contextual understanding and to explain quantitative findings. Integration of both phases enabled complementary interpretation of the magnitude and underlying mechanisms of presenteeism.

### 2.2. Quantitative phase

#### 2.2.1. Participants and sampling in a quantitative study

An online cross-sectional pilot survey was conducted from June 1 to June 30, 2024. The study population (physicians and nurses) was selected using a nonprobability convenience sampling strategy. All participants were recruited from three state referral health facilities, whose names are not provided to ensure the confidentiality of participants. In Mongolia, state referral hospitals represent health facilities that provide specialized and advanced medical care, serving as key components of the national health system for complex diagnosis and treatment. The minimum sample size for this pilot project was estimated at 195 participants. This accounted for an estimated non-response rate of 10% needed to achieve a satisfactory accuracy based on a 95% confidence interval, and a 5% conservative estimated prevalence of presenteeism [10].

The calculation for the pilot research project was as follows:

$$\begin{array}{c} P\left(\chi >0\right)=1-{\left(1-\pi \right)}^{n},\;n=\frac{\ln\left(1-\text{γ}\right)}{\ln\left(1-\text{π}\right)}=\frac{\text{ln}(1-0.95)}{\text{ln}(1-0.05)}=58.40\approx 59;\\ P=\left(59+6\right)*3=195 \end{array}$$ (1)

To maximize participation, all eligible healthcare professionals (N = 890) from the three selected hospitals were invited to participate. A total of 495 responses were received. After excluding incomplete or invalid responses, 465 participants were included in the final analysis, yielding a response rate of 52.2%. All participants provided informed consent.

#### 2.2.2. Quantitative data collection

Prior to data collection, the instrument was translated into the local language by a certified translation service, followed by a back translation by a different bilingual researcher to ensure consistency. Then, the instrument was pre-tested in a sample of 20 participants who were not in the main study population. This pre-test was conducted one week before the main survey, and its findings were used to inform revisions and corrections and to ensure clarity and validity of the questionnaire.

The self-administered questionnaire consisted of 53 items in 5 domains: 11 items on demographics, 7 items on presenteeism that used the Work Functionality (WFun) impairment scale, 6 items for the Health-Related Quality of Life (HRQoL), 23 items from the simplified Brief Job Stress Questionnaire (BJSQ), and 6 items that assessed work and rest balance [11]–[13]. These measures have been previously applied in occupational health research and demonstrated acceptable validity and reliability. All questionnaire items were based on the Japan Medical Association's national survey and the Stress Check Program, with a recall period of four weeks [14]. The questionnaire was translated and culturally adapted following established cross-cultural adaptation guidelines, including forward translation, back-translation, and expert review to ensure semantic and conceptual equivalence. The final questionnaire was administered online via the JotForm platform. The first page provided detailed information about the study objectives, procedures, and rights of participants. Informed consent was obtained electronically and was required before completing the questionnaire.

#### 2.2.3. Quantitative data analysis

The collected data were cleaned and coded in Microsoft Excel (Version 2511 Build 16.0.19426.20186) and analyzed using RStudio (Version 2025.05.0 + 496). Descriptive statistics summarized participant characteristics and key variables. The WFun tool consists of 7 items assessing work functioning impairment, reflecting the degree of impairment in work function. Each item is rated on a 5-point scale, with total scores ranging from 7 to 35; higher scores indicate greater impairment. A cutoff score of >21 indicates significant work functioning impairment, which is reported as the presence of presenteeism [11].

Univariate analysis was used to identify the potential determinants of presenteeism. Each variable from the univariate analysis that had a p-value below 0.2, and sufficient supporting evidence from prior literature was entered into a multivariable logistic regression model [15]. Model selection used the Akaike Information Criterion, and multicollinearity was assessed using the variance inflation factor (VIF) with generalized VIF < 2. Quantitative reporting followed the Strengthening the Reporting of Observational Studies in Epidemiology (STROBE) guidelines [16].

### 2.3. Qualitative phase

#### 2.3.1. Participants and sampling in the qualitative study

Semi-structured individual interviews examined clinician managers' perceptions of presenteeism and its organizational and contextual determinants [17]. The qualitative phase was conducted within an interpretivist paradigm, which assumes that workplace experiences such as presenteeism are socially constructed and best understood through participants' perspectives and contextual interpretations.

A homogenous, purposeful sample was drawn from the quantitative phase participants, from which clinician managers were later selected based on demographic and professional characteristics for the qualitative phase. Clinician managers were defined as medically trained staff with administrative or combined supervisory and clinical responsibilities. Administrative staff at each health facility provided lists of 92 eligible clinician managers, from which participants were selected. The inclusion criteria required that participants (1) currently held a managerial/supervisory role, (2) had at least five years of experience in the Mongolian health sector, and (3) provided informed consent. Exclusion criteria included clinician managers who had less than five years of professional experience in the Mongolian health sector, those not actively working during the study period (e.g., on extended leave), and those unwilling to provide informed consent.

#### 2.3.2. Qualitative data collection

ET conducted face-to-face interviews between February and May 2025, with OO coordinating recruitment. The interviewer had qualitative research training and no supervisory relationship with participants. Reflexivity was applied throughout the qualitative process. The interviewer reflected on their professional background and potential assumptions when conducting interviews. Reflexive memoing was used to document emerging thoughts and decisions during data collection and analysis, and regular discussions among the research team helped critically examine interpretations and minimize individual bias [18]. Interviews took place in private rooms using a semi-structured interview guide (Supplementary Table S2). The guide was developed based on the preliminary findings of the quantitative phase and informed by the Job Demands–Resources Model. Key quantitative results related to workload, staffing shortages, and workplace conditions associated with presenteeism were used to formulate the interview questions, which aimed to explore participants' perspectives and provide deeper contextual explanations of the quantitative findings. Interviews lasted 50–80 minutes, were audio-recorded with consent, and data collection proceeded with concurrent analysis until thematic saturation was reached [19].

#### 2.3.3. Qualitative data analysis

All interviews were transcribed, anonymized, and analyzed in MAXQDA using Braun and Clarke's six-step thematic analysis [20],[21]. Two authors (OB, OC) independently coded the transcripts and developed themes through iterative discussion guided by the JD-R framework. A third author (DD) reviewed the final coding scheme. All transcripts were analyzed in Mongolian, as all five authors involved in the qualitative phase were native Mongolian speakers. Selected quotes were translated into English by OB and verified by DD and OC. Credibility, dependability, confirmability, and transferability were enhanced through member checking, triangulation, audit trails, reflexive memoing, and rich contextual description [22]. Reporting followed the APA reporting standard and the Consolidated Criteria for Reporting Qualitative Research (COREQ) and EQUATOR guidelines [18].

### 2.4. Integration of quantitative and qualitative results

In this explanatory sequential mixed-methods study, quantitative and qualitative findings were integrated through a triangulation approach [23]. Quantitative results informed the subsequent qualitative phase, in which in-depth interviews were conducted to explain and contextualize the findings. Integration was achieved through a joint display table that linked the quantitative results with corresponding qualitative themes, enabling direct comparison. Findings were interpreted through confirmation, expansion, and discordance to identify convergence, provide deeper insights, and highlight areas requiring further investigation.

Ethical approval was obtained from the Research Ethics Board of the Ministry of Health, Mongolia (24/041), and Epidemiological Research Ethics Review Committee of Hiroshima University, Japan (E2025–0118). Written consent was obtained from interviewees under the Research Ethics Board of the Ministry of Health, Mongolia (24/041). The study also adhered to the principles of the Declaration of Helsinki [24].

## 3. Results

### 3.1. Quantitative study

A total of 465 physicians and nurses completed the online survey (Table 1). Most participants were female (87.5%), younger than 40 years of age (61.1%), and married (68.6%), lived in an apartment (68.0%), and reported an average monthly household income of 350,000–750,000 Mongolian Tugrug (63.4%) (USD 98.8–211.6 as of December 10, 2025) per family member. Nurses accounted for a larger proportion of respondents (64.5%) than physicians (35.5%). Over half of the participants had more than 11 years of work experience (51.0%), and most were employed in inpatient care units (63.4%).

Nearly two-thirds of participants (64.5%) reported presenteeism. A multivariable logistic regression analysis with adjustments for confounding (Table 2) demonstrated that three factors were significantly associated with presenteeism: psycho-physical complaints (AOR = 4.51, 95% CI: 2.57–8.26, p < 0.001); difficulties in relationships with coworkers (AOR = 2.38, 95% CI: 1.52–3.80, p = 0.0002); and greater workload (AOR = 2.08, 95% CI: 1.40–3.13, p = 0.0003). Age, marital status, income, occupation (nurse vs. physician), work experience, degree of control over work, support from superiors, sleep duration, night shift frequency, or patient complaints had no significant effects.

### 3.2. Qualitative study

Fourteen clinician managers (three males and eleven females) were recruited from the three health facilities (Table 3). All participants were in charge of a unit and had worked in the managerial role for 6–32 years, with a mean professional experience of 18.7 years in the health sector and 5.9 years in leadership roles. Managerial responsibilities accounted for 66% of their daily work.

Qualitative analysis identified five interrelated themes reflecting the systemic, organizational, and contextual drivers of presenteeism among participants. These themes were (1) inadequate occupational health policy, (2) shortcomings of occupational health infrastructure, (3) staff shortages and heavy workloads, (4) informal workplace practices and professional norms, and (5) physical and mental health burdens. Participants emphasized the need for coordinated action at policy and facility levels to address issues related to presenteeism. The following sections elaborate on each theme, illustrated with participant quotations (Table 4).

*Theme 1. Inadequate occupational health policies*

A central theme from the analysis was the inadequacy of occupational health policies at national and institutional levels. Although formal policies existed, participants reported that they were fragmented, inconsistently implemented, and insufficient to protect healthcare professionals' well-being or optimize professional performance. Moreover, many participants highlighted weak social protection, specifically regarding their salary and incentives (Q1–2). Limited access to standardized paid sick leave was a major concern, with staff often relying on vacation days or unpaid leave to manage illness, reinforcing presenteeism. When facility-based outpatient care was available, they were frequently described as fragmented and ineffective (Q3).

Many participants highlighted insufficient safeguards against workplace harassment and violence, limited or absent social protection mechanisms, and inadequate income security, which constrained their ability to prioritize health, especially amid inflation (Q4). Frequent leadership changes, policy incoherence, weak implementation, and lack of coordination were identified as structural barriers. Institutional cultures that prioritize uninterrupted service often hinder the enforcement of worker protections. Existing initiatives were perceived as poorly grounded, inconsistently implemented, and adding administrative burdens rather than meaningful support (Q5–6).

The majority of participants emphasized the need for robust, evidence-based, and sustainable policies to effectively promote occupational health and well-being. Structural gaps frequently reported by participants create work environments that discourage recovery, exacerbate strain, and perpetuate presenteeism, highlighting systemic challenges beyond individual or interpersonal factors (Q7).

*Theme 2. Shortcomings of occupational health infrastructure*

Many participants reported critical deficiencies in occupational health infrastructure that undermined safe and effective work performance (Q8). Participants noted that rest facilities were inadequate, with limited access to proper rest areas, meal breaks, and bathrooms during shifts (Q9). Night-shift staff were often required to work overtime without relief. Workspaces were frequently overcrowded, poorly maintained, and equipped with outdated furniture and tools. Workplace well-being was widely neglected (Q10).

Most participants emphasized the lack of support for psychological health. There were no stress management programs, routine health monitoring, or staff health surveillance, leaving mental well-being largely unrecognized (Q11).

Participants also highlighted that a lack of essential equipment further hindered productivity. Outdated instruments and the need to purchase basic supplies personally were frequently mentioned (Q12).

*Theme 3. Staff shortages and heavy workloads*

Participants consistently reported that persistent staff shortages and escalating workloads undermine healthcare quality, worker well-being, and performance (Q13). Key contributors included population growth, rising disease incidence, outdated workforce planning, and high turnover driven by workload pressures, work–life imbalance, limited incentives, and low salaries (Q14).

Most participants noted that underskilled or inexperienced staff further increased the burden on qualified personnel. Excessive workloads resulted from disorganized task allocation, uneven distribution of responsibilities, and limited leadership support (Q15). Capacity overload relative to patient demand, insufficient consultation time, and multitasking expectations compounded these challenges. Political instability was also described as disrupting institutional continuity, adding to staff strain (Q16).

Participants highlighted a cyclical relationship between staff shortages and workload, each reinforcing the other and contributing to attrition (Q17).

*Theme 4. Informal workplace practices and professional norms*

Participants described entrenched informal practices and professional norms that shaped the work environment, often in ways that were burdensome, inequitable, and misaligned with formal employment expectations. Overtime and work-related activities outside regular hours were commonly expected, typically without additional compensation. Unwritten rules required staff to work despite illness, reflecting an unhealthy work culture (Q18–19).

Many participants reported pressure from supervisors and colleagues to take on tasks beyond their scope, with highly committed staff disproportionately assigned additional duties. Supervisors, often physicians, were frequently criticized for poor management and leadership. Generational conflicts were also noted, including differences in values, workplace attitudes, and mutual respect, which contributed to strained professional relationships (Q20–21).

*Theme 5. Physical and mental health burdens*

Participants consistently reported substantial physical and psychological strain that impaired both daily functioning and professional performance. Many continued working despite illness or distress, frequently neglecting their own health. Commonly reported conditions included metabolic and gastrointestinal disorders (e.g., diabetes, pancreatic issues), musculoskeletal pain, kidney problems, allergies, and, in some cases, cancer (Q22–23). Participants also highlighted frequent exposure to infectious diseases, including influenza and MRSA. Across all age groups, somatic symptoms, including fatigue, headaches, sleep disturbances, neurological complaints, palpitations, arrhythmias, hypertension, and other cardiovascular problems, were frequently described, often linked to chronic stress (Q24–25).

High job demands, combined with unmet physical and social needs, were reported to exacerbate stress, anxiety, depression, and burnout. Despite their impact on health and occupational performance, these conditions were often underrecognized or deprioritized by both institutional leadership and the healthcare professionals themselves (Q26–28).

### 3.3. Integrated results

In this explanatory sequential mixed-methods study, integration of results was conducted using a triangulation approach and presented through a joint display (Table 5), linking quantitative results with corresponding qualitative themes. The integration focused on identifying areas of (1) confirmation, where findings converged; (2) expansion, where qualitative data provided deeper explanations of quantitative associations; and (3) complementarity, where qualitative findings revealed unmeasured structural factors.

The quantitative analysis identified psycho-physical complaints, coworker relationships, and workload as significant factors associated with presenteeism. These findings were further explained and contextualized by qualitative results. First, the strong association with psycho-physical complaints was supported by qualitative evidence describing chronic physical and psychological strain, with participants frequently reporting that healthcare professionals continue working despite illness due to both internalized professional responsibility and external pressures. Second, the association with coworker relationships was clarified through qualitative findings highlighting informal workplace norms and peer expectations. Participants described a culture in which taking sick leave is perceived as a burden on colleagues, creating implicit pressure to attend work even when unwell. Third, the effect of workload was explained by systemic issues, including persistent staff shortages, uneven task distribution, and high patient demand. These structural constraints limit opportunities for rest or recovery and reinforce presenteeism as a coping mechanism. Additionally, qualitative findings identified organizational and policy-level factors not directly captured in the quantitative model, including inadequate occupational health infrastructure and weak policy implementation. These findings expand the interpretation of quantitative results by situating individual-level associations within broader systemic conditions.

Overall, the integration shows that presenteeism arises from the interaction of individual health burdens, workplace norms, workload pressures, and structural constraints within the healthcare system. This approach enabled a more comprehensive understanding of both the magnitude and underlying mechanisms of presenteeism.

**Table 1.** Prevalence of presenteeism by sociodemographic characteristics.

Characteristic	Overall (N = 465)		Presenteeism			
			Absent (N = 165; 35.5%)		Present (N = 300; 64.5%)	

	N	Row %	N	Column %	N	Column %
Gender						
Male	58	12.5%	23	39.7%	35	60.3%
Female	407	87.5%	142	34.9%	265	65.1%
Age						
≤30	137	29.5%	51	37.2%	86	62.8%
31–40	147	31.6%	58	39.5%	89	60.5%
41–49	115	24.7%	36	31.3%	79	68.7%
50–59	66	14.2%	20	30.3%	46	69.7%
Marriage status						
Married	319	68.6%	100	31.3%	219	68.7%
Not married/cohabitating	118	25.4%	54	45.8%	64	54.2%
Separated/divorced/widowed	28	6.0%	11	39.3%	17	60.7%
Average monthly household income per family member (MNT)*						
<350,000	89	18.9%	39	43.2%	50	56.8%
350,000–750,000	295	63.4%	97	32.9%	198	67.1%
>750,000	81	17.4%	29	35.8%	52	64.2%
Dwelling						
Apartment	316	68.0%	112	35.4%	204	64.6%
House (Ger district)	77	16.6%	28	36.4%	49	63.6%
Ger (Ger district)	30	6.5%	10	33.3%	20	66.7%
Public/communal housing	19	4.1%	10	52.6%	9	47.4%
Private house	6	1.3%	2	33.3%	4	66.7%
Other	17	3.7%	3	17.6%	14	82.4%
Working years						
≤5 years	125	26.9%	46	36.8%	79	63.2%
6–10 years	103	22.2%	43	41.7%	60	58.3%
11–20 years	136	29.2%	49	36.0%	87	64.0%
≥21 years	101	21.7%	27	26.7%	74	73.3%
Occupation						
Physician	165	35.5%	68	41.2%	97	58.8%
Nurse	300	64.5%	97	32.3%	203	67.7%
Working unit						
Inpatient clinic	295	63.4%	108	36.6%	187	63.4%
Outpatient clinic	106	22.8%	35	33.0%	71	67.0%
Other	64	13.8%	22	34.4%	42	65.6%

Note: *MNT, Mongolian tugrug.

**Table 2.** Multivariable logistic regression for factors associated with the presence of presenteeism.

Predictor	AOR	95% CI		P-value

		Lower	Higher	
Age				
Under 40 years old	Reference			
Over 40 years old	1.62	0.86	3.13	0.1421
Marriage status				
Not married	Reference			
Married	1.26	0.75	2.10	0.3845
Income				
<350,000	Reference			
350,000–750,000	0.70	0.38	1.30	0.2560
>750,000	0.74	0.34	1.60	0.4464
Occupation				
Physician	Reference			
Nurse	0.71	0.42	1.20	0.1997
Working years				
Under 10 years of working experience	Reference			
Over 10 years of working experience	0.75	0.40	1.40	0.3666
Work factors				
Quantity workload	2.08	1.40	3.13	0.0003
Degree of control	0.98	0.65	1.49	0.9365
Psycho-physical complaints	4.51	2.57	8.26	0.0000
Superiors	1.27	0.85	1.89	0.2335
Coworkers	2.38	1.52	3.80	0.0002
On-call days				
Zero	Reference			
More than once	1.20	0.74	1.92	0.4607
Sleep duration				
More than 5 hours of sleep	Reference			
Less than 5 hours of sleep when not working on the night shift	1.44	0.88	2.40	0.1541
Night-shift days				
Zero	Reference			
Had a night shift more than one time in the past month	1.03	0.61	1.73	0.9241
Complaints from the patient and their family				
None	Reference			
Received complaints more than once in the past 6 months	1.40	0.80	2.42	0.2348

**Table 3.** Characteristics of the qualitative study participants.

Participant ID	Work experience in the health sector (years)	Leadership role (years)	Type of unit
P01	12	2	Outpatient unit
P02	21	6	Inpatient unit
P03	6	1	Patient support unit
P04	32	10	Outpatient unit
P05	23	10	Inpatient unit
P06	25	8	Medical services unit
P07	21	5	Patient support unit
P08	21	7	Medical services unit
P09	12	7	Patient support unit
P10	25	5	Medical services unit
P11	23	15	Patient support unit
P12	13	1	Outpatient unit
P13	12	3	Inpatient unit
P14	16	2	Inpatient unit

**Table 4.** Representative quotes.

Inadequate occupational health policy
**Q1.** “In Mongolia, laws, regulations, and policies exist on paper, but they are not fully implemented. Everything comes in with a fancy title, but in reality, it's disconnected. Frequent changes in leadership disrupt continuity. Orders change with leadership, and there is no stability or real protection for health workers.” (**P10**)
**Q2.** “Our salaries and incentives are not enough to survive. We cannot prioritize our health when we are worried about loans, rent, and daily survival. There is almost no one without loans.” (**P7**)
**Q3.** “Healthcare workers are expected to work even when sick. There is no real paid sick leave. People keep working because there is no one to replace us.” (**P8**)
**Q4.** “Patients insult, threaten, and even attack staff, yet there is no legal protection for us from harassment or violence. Complaint systems punish workers rather than protect them. Even when workers are clearly mistreated, they are required to apologize or attend disciplinary hearings.” [...] “When staff raise concerns, they are told, ‘You took an oath, so do your job without complaining.’ This reflects a culture that discourages workers from prioritizing their own health.” (**P12**)
**Shortcomings of occupational health infrastructure**
**Q5.** “We don't have proper places to rest, eat, or even use the bathroom during long shifts. Night shifts go on without relief. (**P3**)
**Q6.** “Workspaces are overcrowded, poorly maintained, and constantly under repair. The physical environment itself creates frustration.” [...] “We work with outdated computers and equipment, and sometimes even buy basic supplies with our own money. This slows work and increases frustration.” (**P9**)
**Q7.** “There are no mental health management programs, no psychological monitoring, and no trained psychologists for staff. Psychological well-being is completely overlooked.” (**P4**)
**Staff shortages and heavy workloads**
**Q8.** “There are too few staff for the number of patients. Patient numbers keep increasing, but staffing remains the same, so everyone is overloaded. Staff shortages and workload feed into each other.” [...] “Low salaries, long hours, and burnouts push people to quit or look for other options.” (**P5**)
**Q9.** “Doctors see far more patients than standards allow. There is no time to rest or communicate properly, and multitasking all day hinders care quality.” (**P8**)
**Q10.** “When inexperienced staff are assigned, the workload on experienced workers doubles. We must do our own work and also supervise, correct mistakes, and take responsibility.” (**P10**)
**Informal workplace practices and professional norms**
**Q11.** “There is an unwritten rule that healthcare workers have no right to be sick. Taking time off is seen as letting the team down.” (**P11**)
**Q12.** “Overtime and work outside regular hours or calls on our days off are simply expected, usually without any compensation. Personal time with family is constantly interrupted” (**P12**)
**Q13.** “Highly committed staff are always given more work beyond the job descriptions. Many supervisors are doctors without management training. They rely on authority, not coordination, and this creates miscommunication and tension within teams.” (**P13**)
**Q14.** “There are growing conflicts between generations. Differences in values, work ethic, and respect strain teamwork and morale.” (**P6**)
**Physical and mental health burdens**
**Q15.** “Over the years, this job itself makes people sick. We develop diabetes, high blood pressure, kidney and thyroid problems, back pain, and even cancer. We keep working, and by retirement, almost everyone has serious health problems.” (**P2**)
**Q16**. “Chronic and infectious diseases are very common, even among young staff.” (**P14**)
**Q17.** Many of us are emotionally exhausted. Anxiety, burnout, and depression are everywhere, but people don't always realize how severe it is until they break down or make mistakes.” [...] “We absorb and endure patients' stress every day. Even when exhausted or ill, we must perform perfectly because lives depend on us.” (**P1**)

**Table 5.** Integrated results through triangulation.

Quantitative evidence	Qualitative evidence	Themes	Triangulation insight
Psycho-physical complaints AOR = 4.51, p < 0.001	Physicians and nurses push through chronic physical and mental strain	Physical and mental health burdens	Strong alignment: Quantitative data supported by qualitative evidence
Coworker support AOR = 2.38, p = 0.002	Peer dynamics and expectations sustain a culture of showing up, even when unwell	Informal workplace practices and professional norms	Consistent: Peer support is quantitatively linked to presenteeism, qualitatively explained by workplace norms and pressure.
Superiors' support AOR = 1.03, p = 0.908	Leadership support lacks consistency or enforcement		Quantitative data show no significant effect, but qualitative data suggest inconsistent leadership support.
Quantity of workload AOR = 2.08, p = 0.0005	Due to understaffing and high demands, staff feel compelled to work regardless of their condition	Staff shortages and heavy workloads	Clear link: Heavy workload is quantitatively significant, qualitatively linked to staff shortages and workload pressures.
Not directly measured; indirectly captured through work–life interference and complaints	Inadequate rest spaces, breaks, and policies discourage recovery	Shortcomings in occupational health infrastructure	Complementary: Highlights unmeasured dimensions of working through harmful work conditions.
No policy-specific items or direct measures	Broader structural gaps, such as weak policies and inconsistent implementation	Inadequate occupational health policy	Supplementary: A systemic weakness clearly emerging from interviews, calling for policy-level intervention.

## 4. Discussion

Using an explanatory sequential mixed-methods design, including a pilot quantitative phase followed by qualitative inquiry, we conducted a cross-sectional web-based survey among physicians and nurses in mid-2024, followed by interviews with clinician managers in early 2025. Presenteeism among Mongolian healthcare professionals emerged as a structurally embedded phenomenon driven by psycho-physical health complaints, informal workplace norms, staff shortages, and gaps in occupational health infrastructure and policy. The relatively narrow confidence intervals for key variables (e.g., psycho-physical complaints and workload) indicate stable estimates, whereas wider intervals for nonsignificant variables suggest greater uncertainty. Several variables were not significantly associated with presenteeism after adjustment. This may reflect limited independent effects or mediation by stronger predictors, such as psycho-physical complaints and workload, which demonstrated stronger associations in the model.

Quantitatively, 64.5% of participants reported moderate-to-severe work impairment, exceeding the pooled global estimate (49.2%) and many country-level reports [14],[25], though comparable to China (66.40%) [26]. These variations may reflect differences in measurement tools and healthcare system contexts. Qualitative findings identified five interconnected drivers of presenteeism: inadequate occupational health policies, shortcomings in occupational health infrastructure, staff shortages and heavy workloads, informal workplace practices and professional norms, and physical and mental health burdens. Together, these findings highlight how systemic conditions shape organizational environments and individual health behaviors.

Inadequate occupational health policies and weak enforcement exacerbate presenteeism [27]. Limited access to paid sick leave, financial constraints, and burdensome documentation [28] discourage workers from taking sick leave [29]. More broadly, weak legal and social protections, low salaries [6], exposure to violence and harassment [30], and financial insecurity [31] compel health professionals to continue working despite illness [32]. These policy gaps are compounded by political instability, bureaucratic delays, leadership turnover, and competing institutional priorities, which undermine consistent policy implementation [30]. As a result, healthcare facilities often prioritize uninterrupted service delivery over worker protection, fostering normalized presenteeism. Existing initiatives are frequently perceived as symbolic or administratively burdensome rather than supportive [30].

Furthermore, shortcomings in the occupational health infrastructure intensify presenteeism. Insufficient rest areas and time, outdated equipment, lack of monitoring, and neglect of worker well-being force professionals to work under unsafe and exhausting conditions [5],[33]. In the Mongolian health sector, inadequate equipment and outdated tools also prevent the effective delivery of services and force employees to work under unsatisfactory conditions [5],[34]. These deficiencies reduce productivity, compromise care quality [30], and create strain, reinforcing presenteeism [8]. Staff shortages and heavy workload were also central drivers. Despite workforce density exceeding WHO thresholds [35], geographic maldistribution has led to shortages in rural and specialized settings [5],[34]. Low pay, inadequate social protection, and high turnover further intensify workloads [6],[31], disrupt work–life balance [30], and contribute to attrition [5],[7], highlighting the need for better workforce management [36]. Informal workplace practices and professional norms encourage presenteeism. Strong professional obligations, authoritarian leadership styles, and limited managerial capacity discourage the use of sick leave [29],[36]. Generational differences within the workforce can further strain team dynamics, morale, and organizational culture [29],[30]. Consistent with previous research, physical and mental health burdens strongly contributed to presenteeism [4],[26],[28]. Participants reported chronic conditions [37], frequent infections [2], and stress-related symptoms linked to demanding work conditions, shift work, and sustained occupational stress [8].

Overall, our findings align with recent occupational health models framing presenteeism as a structural phenomenon shaped by institutional norms, leadership practices [36], and policies [1],[25]. Addressing it requires systemic reform, including evidence-based, context-sensitive policies, stronger occupational health governance, supportive leadership, and sustained resource allocation.

This is the first mixed-methods study to examine presenteeism among physicians and nurses in Mongolia, addressing a critical evidence gap. Integration of quantitative and qualitative data and inclusion of multiple urban and rural hospitals strengthen the findings. The findings suggest the need for multi-level interventions, including improved working conditions, organizational support, and policies that promote workforce well-being and the use of appropriate sick leave. However, several limitations should be considered. First, the quantitative component of this study was conducted as a pilot survey; therefore, the sample size was not designed to provide nationally representative or fully generalizable estimates. Accordingly, the quantitative findings should be interpreted as exploratory and hypothesis-generating rather than confirmatory. Second, the study was limited to three state hospitals and used a nonprobability sampling approach, which may introduce selection bias. Third, the reliance on self-reported, cross-sectional data may be subject to reporting bias and limit causal inference. Fourth, some variables included in the regression model showed no significant association after adjustment, which may reflect limited independent effects, measurement constraints, or mediation by stronger predictors. In addition, residual confounding from unmeasured factors cannot be excluded. Fifth, the qualitative component included only clinician managers, which may not fully capture the perspectives of frontline healthcare workers. Finally, although the WFun tool also has not yet been validated in Mongolia, validation is currently underway. This tool, designated for assessing work functioning impairment, does not directly measure the behavior of attending work while ill; it is widely used to capture its functional consequences in occupational health research. Therefore, building on recent conceptual advances, future studies should integrate direct measures of presenteeism behavior with indicators of work functioning impairment to better capture both the occurrence and consequences of working while ill [38]. Despite these limitations, the use of an explanatory sequential mixed-methods design and the integration of quantitative and qualitative findings through triangulation strengthen the credibility of the results by providing both preliminary patterns and in-depth contextual explanations.

## 5. Conclusions

This study provides novel evidence that presenteeism among Mongolian healthcare professionals is a systemic issue shaped by organizational practices, institutional culture, and policy gaps. The findings highlight that both individual- and system-level factors contribute to the persistence of presenteeism, with important implications for workforce well-being and quality of care. Addressing this issue requires coordinated, evidence-based interventions, including improvements in working conditions, strengthening organizational support, and the development and enforcement of policies that promote appropriate sick leave use and occupational health protections. Such efforts are essential to enhance healthcare system resilience, safeguard healthcare professionals' well-being, and maintain high-quality patient care in Mongolia and similar resource-constrained settings.

## Use of AI tools declaration

The authors declare they have not used Artificial Intelligence (AI) tools in the creation of this article.


