# Supplementary material for: Presenteeism among healthcare professionals in Mongolia: A mixed-method multicenter study
Source: AIMS Public Health. 2026 Jul 13;13(3):741–60. doi: 10.3934/publichealth.2026040 (PMC13684083; doi:10.3934/publichealth.2026040)
Supplement: Supplementary file 1 [file publichealth-13-03-040-s001.pdf]

Public Health

AIMS Public Health, 13(3): 741–760.
DOI: 10.3934/publichealth.2026040
Received: 23 January 2026
Revised: 10 April 2026
Accepted: 06 May 2026
Published: 13 July 2026

http://www.aimspress.com/journal/aimsph

Research article

Presenteeism among healthcare professionals in Mongolia: A mixed-method multicenter study

Oyundari Batsaikhan[1], Odgerel Chimed-Ochir[1,*], Delgerjargal Dorjbal[2], Erdmaa Tserendondov[3], Otgontogoo Oidovsuren[3], Yumiya Yui[1], Ami Fukunaga[1], Yoshihisa Fujino[4], Bayasgalanmunkh Baatar[3] and Tatsuhiko Kubo[1]

[1] Department of Public Health and Health Policy, Hiroshima University, Hiroshima, Japan
[2] Department of Rehabilitation Medicine, Fujita Health University, Toyoake, Aichi, Japan
[3] Darkhan-Uul Medical School, Mongolian National University of Medical Sciences, Darkhan-Uul province, Mongolia
[4] Department of Environmental Epidemiology, University of Occupational and Environmental Health, Kitakyushu, Japan

* **Correspondence:** Email: odgerel@hiroshima-u.ac.jp; Tel: +81822575169.

Supplementary

Supplementary Table S1. Explanatory sequential mixed-methods approach of this study, starting with the quantitative phase, followed by the qualitative phase, and findings integration.

	Quantitative analysis	Qualitative analysis
Participants	• Physicians and nurses	• Clinician managers
Sampling	• Nonprobability sampling (N = 465 physicians and nurses)	• Purposeful sampling (N = 14 clinician managers)
Data collection instruments	Data collection tool<br>• Cross-sectional, web-based questionnaire consisting of questions on:<br>- Demographics–11,<br>- Work functionality impairment tool (WFun)–7<br>- Health-related quality of life (HRQOL)–6<br>- Simplified brief job stress questionnaire (BJSQ)–23<br>• Informed consent	• Semi-structured interviews addressing:<br>- Perception and experience of presenteeism<br>- Currently implemented measures<br>- Further needs<br>• Informed consent<br>• Audio recording<br>• Transcription<br>• Translation
Data analysis	• Descriptive analysis<br>• Univariate and multivariate regression analysis	• Thematic analysis<br>- Familiarization with data<br>- Generating initial codes<br>- Searching for themes<br>- Reviewing themes<br>• Defining and naming themes<br>Lincoln and Cuba's trustworthiness criteria
Findings integration	• Integration of the quantitative and qualitative results<br>• Methodologic and investigators triangulation	

Supplementary Table S2. A semi-structured interview guide.

Domain	Main questions	Probing questions
1. Work context	• Can you briefly describe a typical day in your role as a clinician manager?	- What is your role, and approximately how many personnel are you responsible for?
2. Presenteeism experiences	• How common is presenteeism at your workplace?	- Can you describe situations when staff continue to work despite illness?
3. Job demands	• What are the main challenges at work that affect presenteeism?	- Which aspects of your work feel most demanding (e.g., workload, emotional demands, role conflict, time pressure)?<br>- Are there any unspoken expectations or informal norms that influence whether staff work while ill?
4. Job resources	• What supports are available at your workplace to manage demands and reduce presenteeism?	- What supports are lacking or insufficient?<br>- How do relationships with colleagues, supervisors, or the organization influence your ability to cope with work demands?
5. Personal resources	• What personal strengths or strategies help you manage work while experiencing illness?	- What personal resources help mitigate individual risk factors for presenteeism?
6. Outcomes	• How do the mentioned demands and resources affect presenteeism?	- In your opinion, what are the main causes of presenteeism (e.g., physical and mental health, work-related factors)?<br>- How do these factors affect your well-being, work performance, and patient care?
7. Improvements and future directions	• How could current measures to prevent or reduce presenteeism be strengthened in Mongolia?	- In your opinion, what should be the priority of interventions at your organization and at the health system level?<br>- How do you think measures to reduce or prevent presenteeism will evolve in the future?
8. Closing question	• Is there anything else you would like to add regarding presenteeism or related workplace challenges?	

AIMS Press

© 2026 the Author(s), licensee AIMS Press. This is an open access article distributed under the terms of the Creative Commons Attribution License (http://creativecommons.org/licenses/by/4.0)
